# Raman micro-spectroscopy as a tool to study immunometabolism

**DOI:** 10.1042/BST20230794

**Published:** 2024-03-13

**Authors:** Jiabao Xu, Karl J Morten

**Affiliations:** 1 Division of Biomedical Engineering, James Watt School of Engineering, University of Glasgow, Glasgow G12 8LT, U.K.; 2 Nuffield Department of Women's and Reproductive Health, University of Oxford, The Women Centre, John Radcliffe Hospital, Headley Way, Headington, Oxford OX3 9DU, U.K.

**Keywords:** cell sorting, imaging techniques, immunometabolism, Raman spectroscopy

## Abstract

In the past two decades, immunometabolism has emerged as a crucial field, unraveling the intricate molecular connections between cellular metabolism and immune function across various cell types, tissues, and diseases. This review explores the insights gained from studies using the emerging technology, Raman micro-spectroscopy, to investigate immunometabolism. Raman micro-spectroscopy provides an exciting opportunity to directly study metabolism at the single cell level where it can be combined with other Raman-based technologies and platforms such as single cell RNA sequencing. The review showcases applications of Raman micro-spectroscopy to study the immune system including cell identification, activation, and autoimmune disease diagnosis, offering a rapid, label-free, and minimally invasive analytical approach. The review spotlights three promising Raman technologies, Raman-activated cell sorting, Raman stable isotope probing, and Raman imaging. The synergy of Raman technologies with machine learning is poised to enhance the understanding of complex Raman phenotypes, enabling biomarker discovery and comprehensive investigations in immunometabolism. The review encourages further exploration of these evolving technologies in the rapidly advancing field of immunometabolism.

## Immunometabolism: a decade of dynamic research

In the last two decades, immunometabolism has blossomed into a dynamic and expansive realm of research, offering profound insights into the molecular intricacies of health and disease [1–4]. This field delves into the intricate interplay between cellular metabolism and immune function, spanning diverse cell types, tissues, and diseases. Its implications extend far into therapeutic interventions, significantly enriching our comprehension of the immune-metabolic axis.

A good starting point in investigating cellular metabolic rewiring is Extracellular Flux Analysis, employing the Agilent Seahorse XF analyzer [3]. This approach concurrently measures two energetic pathways, oxidative phosphorylation and glycolysis, providing a comprehensive understanding of metabolic shifts. The advent of metabolomics has been revolutionary, enabling the simultaneous measurement of tens to thousands of metabolites from various sources such as cells, plasma, urine, and tissues. Platforms like liquid chromatography–mass spectrometry, gas chromatography–mass spectrometry, and NMR have facilitated the extraction, identification, and quantification of metabolites, through either targeted metabolomics to quantify desired targeted molecules or untargeted metabolomics for discovery [5].

Despite these advancements, current approaches to study immunometabolism lack single-cell resolution, and population-based platforms may inadvertently overlook variations in subcellular compartments, potentially compromising the accurate understanding of metabolism. Single-cell metabolomics, while still considered premature for large-scale immunometabolism studies, remains pivotal for mapping metabolic landscapes. As the field of immunometabolism continues to evolve, there is a compelling need to explore additional methodologies with single-cell resolution, addressing limitations and enhancing precision for a more comprehensive characterization.

## Raman micro-spectroscopy unveiled: illuminating metabolism at a microscale

### Raman spectroscopy in biological systems

**Figure 1.**
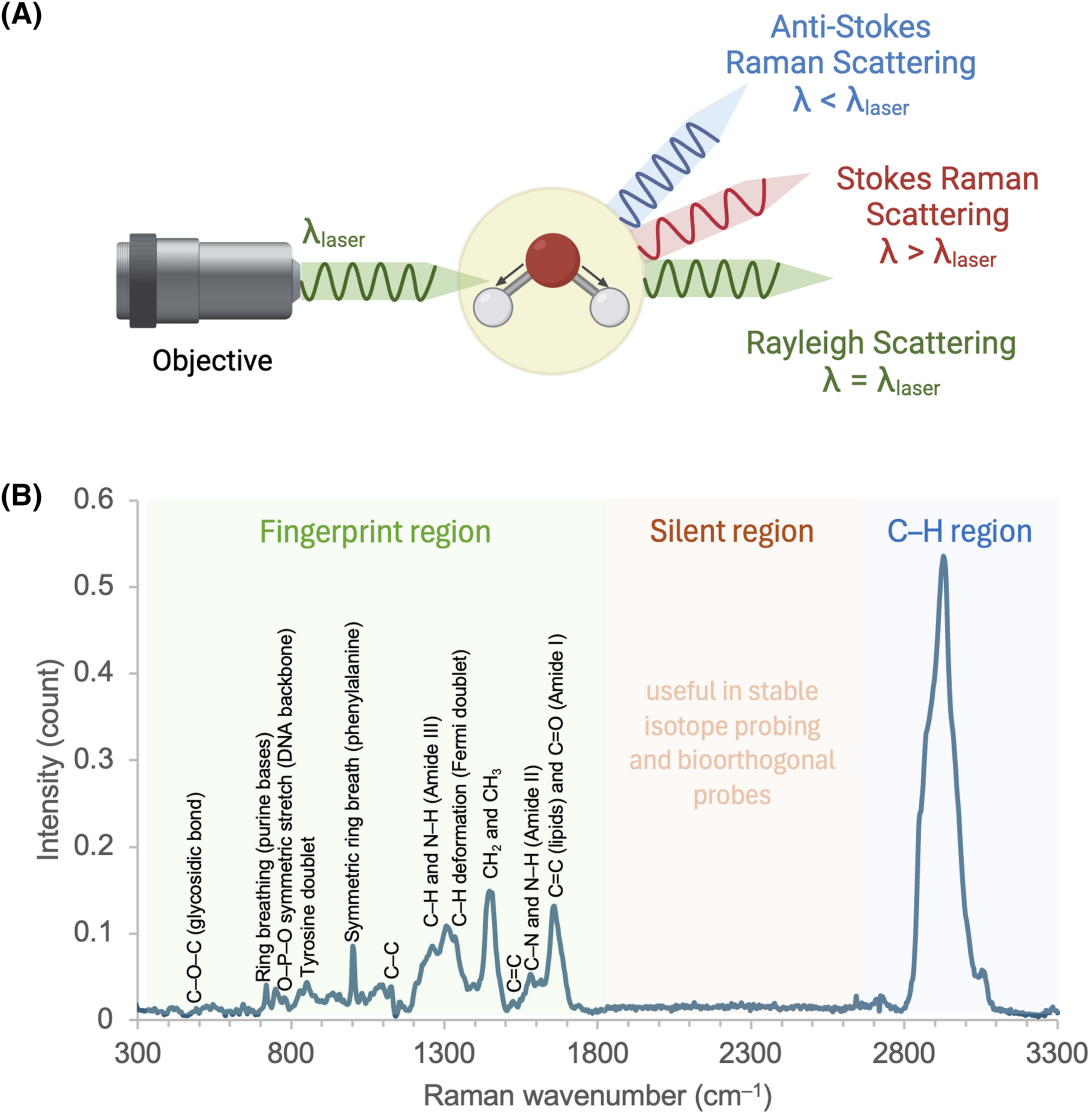
Raman spectroscopy working principles and a typical biological Raman spectrum. (**A**) Three distinct scattering signals produced from the interaction between light and a vibrating molecule. In Rayleigh scattering, the wavelength of the light remains unchanged; conversely, in Raman scattering, the scattered light's wavelength can either increase (Stokes scattering) or decrease (anti-Stokes scattering) compared with the incoming light. (**B**) A typical Raman spectrum of a cell. The molecular composition can be identified from the individual bands, corresponding to different vibrational modes.

Raman spectroscopy is a fundamental research tool utilized across diverse scientific domains, including chemistry, physics, biology, and materials science. The technique involves illuminating a sample with a monochromatic laser beam, generating both elastic (Rayleigh) and inelastic (Raman) scattering upon interaction with molecules (Figure 1A). While elastic scattering dominates, inelastic Raman scattering occurs due to energy exchange between incident photons and molecular vibrations and can be used to probe intrinsic molecular vibrations of a sample. With the advances in optical magnification, Raman micro-spectroscopy is gaining popularity among biologists to look at tissues, cells and even single bacterial cells. Raman micro-spectroscopy offers a unique biological phenotype, showing a comprehensive collection of molecular vibrational profiles within a biological system. In biological studies, it boasts advantages such as high spatial resolution, the ability to detect aqueous samples, intrinsic and label-free characterization, non-contact and non-destructive analysis, and straightforward preparation with small sample volumes.

Despite its potential, the application of Raman spectroscopy in biomedical research presents challenges due to the weak nature of the Raman effect and the significant background noise, predominantly from fluorescence, which can substantially obscure the Raman signal. Furthermore, the complexity of interpreting overlapping Raman bands from biological molecules requires advanced analysis methods, such as demixing and reconstruction, to accurately identify molecular components. These steps are crucial for extracting precise molecular signatures and ensuring reliable results, as further elaborated in section ‘Raman imaging’.

A biological Raman spectrum comprises three distinct regions: the ‘fingerprint’ region (400–1800 cm^−1^), containing essential bio-information and recognized as a sample's fingerprint; the ‘silent’ region (1800–2700 cm^−1^), often lack of vibrational modes from naturally occurring isotopes and can be used for targeted probing of metabolic pathways by stable isotope labeled substrates; and the high-wavenumber region (2700–3200 cm^−1^), primarily contributed by stretching vibrations of C–H groups, notably from lipids and proteins (Figure 1B).

Recognizing the challenge of navigating exhaustive tables of Raman biological assignment in most current studies and reviews, this mini-review streamlines Raman assignments into separate tables for major cellular components: proteins (Table 1), nucleic acids (Table 2), lipids (Table 3), carbohydrates (Table 4), and resonant molecules (Table 5).

**Table 1.** Raman assignment table of amino acids and proteins

**Wavenumber/cm^−1^**	**Biological assignment**	**Vibrational assignment**	**Comments**
510	S–S bridge	ν(S–S) stretch	Proteins with sulfide bridges
620	Phenylalanine	τ(C–C) twist	
643	Tyrosine	τ(C–C) twist	
667	Cystine	ν(C–S) stretch	
830	Tyrosine (s)	Ring breath	Aromatic AA Tyrosine doublet
855	Tyrosine (w)	Ring breath	
924–943	Proline valine	Skeletal C–C stretch & protein backbone (α-helix)	
1000	Phenylalanine (s)	Phe symmetric ring breath	Aromatic AA
1030	Phenylalanine (w)	C–H in plane bend	
1230–1300	Amide III	40% C–H stretch 30% N–H bend	
1235–1240	Amide III		Exist in β-sheet and random coil; absence in α-helix
1340	Tryptophan	C–H deformation	Aromatic AA Fermi doublet
1360	Tryptophan	C–H deformation	
1480–1580	Amide II	60% N–H bend 40% C–N stretch	
1600–1690	Amide I	80% ν(C=O)	
1660–1670	Amide I β sheet		β-sheet
2890	Aliphatic AA	ν(C–H)	
2930	Aliphatic AA & Aromatic AA	ν(C–H)	Proteins main C–H
3060	Aromatic AA	ν(C–H)	
3100	Amide B	N–H stretch	
3500	Amide A	N–H stretch	

**Table 2.** Raman assignment table of nucleic acids

**Wavenumber/cm^−1^**	**Biological assignment**	**Vibrational assignment**	**Comments**
617	Thymine	Ring breathing	Pyrimidine bases
650	Guanine	Ring breathing	Purine bases
670	DNA	Thymine, Guanine	
723	Adenine	Ring breathing	Purine bases
740	Thymine	Ring breathing	
785	DNA/RNA	Ring breathing of C, T and U; O–P–O symmetric stretch	Nucleic acid backbone
790	Uracil	Ring breathing	Pyrimidine bases
790	Cytosine	Ring breathing	Pyrimidine bases
828	DNA/RNA	O–P–O stretch	Nucleic acid backbone
1095	DNA/RNA	Symmetric stretch of ${\mathrm{PO}}_{2}^{-}$	Nucleic acid backbone
1235	Uracil		
1275	DNA	Cytosine, Guanine, Adenine	
1376	DNA	Thymine, Guanine, Adenine	
1490	DNA	Guanine, Adenine	
1578	DNA	Guanine, Adenine	
1671	DNA	ν(C=O) thymine, guanine, cytosine	
2970	Nucleic acids	ν(C–H)	Nucleic acid main C–H

**Table 3.** Raman assignment table of fatty acids and lipids

**Wavenumber/cm^−1^**	**Biological assignment**	**Vibrational assignment**	**Comments**
1060, 1098, 1128	Saturated FA	ν(C–C)	Saturated FA
1265	Unsaturated FA	δ(=C–H)	Intensity increases with increasing double bonds
1295/1300	FA	δ/ω(CH_2_)	Sharp in saturated FA
1080	FA	Skeletal C–C in gauche conformation	All lipids
1440	FA	δ(CH_2_, CH_3_)	All lipids
1655	Unsaturated FA	ν(C=C)	Sharp in UFA and fats; intensity increases with increasing double bonds
1735	Fats	ν(C=O)	Lipids with ester group e.g. cholesteryl esters, phospholipids
2850	Lipids	ν(C–H)	Lipids main C–H
3010	Unsaturated lipids	ν(=C–H)	TUFA/TFA: I1650/I1440

FA, fatty acids; TUFA/TFA, total unsaturated fatty acids/total fatty acids.

**Table 4.** Raman assignment table of carbohydrates

**Wavenumber/cm^−1^**	**Biological assignment**	**Vibrational assignment**	**Comments**
484	Glycogen; polysaccharide	ν(C1–O–C4) glycosidic bond	Glycogen linkage
856	Glycogen; polysaccharide	ν(C1–O–C4); ring breath	
940	Glycogen; polysaccharide	ν(C1–O–C6)	
1050	Glycogen; polysaccharide		
1084	Glycogen; polysaccharide		
1130	Glycogen; polysaccharide		
407	Glucose	Skeletal mode	
424	Glucose	Skeletal mode	
518	Glucose	Skeletal mode	
542	Glucose	Ring deformation	
843	Glucose	ν(C–O–C)	
914	Glucose	Ring vibration	

**Table 5.** Raman assignment table of biomolecules that have absorption bands that resonant with a 532-nm Raman laser

**Wavenumber/cm^−1^**	**Biological assignment**	**Vibrational assignment**	**Comments**
750	Cytochromes	ν(pyr breathing); ν(C_α_C_β_)	Heme proteins such as hemoglobin, myoglobin, cytochromes, and peroxidase.
1128	Cytochromes	ν(C_α_N)	
1298–1312	Cytochromes	ν(C_α_C_β_)	
1585	Cytochromes	ν(C_α_C_m_)_asym_	
1003	β carotene; carotenoid	Ring breathing	Orange pigments
1156	β carotene; carotenoid	ν(C–C) ν(C–H)	
1515	β carotene carotenoid	ν(C=C)	

As shown in Table 1, the three Amide signals are of primary interest for identifying different protein backbone conformations: Amide I (C=O stretching vibration), Amide II and Amide III, both involving coupled C–N stretching and N–H bending vibrations of the peptide group. Besides protein backbones, Raman spectroscopy can also identify amino acids through their side chain modes, such as the Fermi doublet at 1360/1340 cm^−1^ for tryptophan, or the 860/833 cm^−1^ doublet for tyrosine (Table 1). The most significant bands from nucleic acids, found at 1084 and 785 cm^−1^, are presented in Table 2, along with bands corresponding to vibrations of individual nucleotide bases. Table 3 lists Raman bands associated with lipids, which play a vital role in cellular signaling, energy storage, and membrane construction. The most prominent lipid bands are located near 1440 cm^−1^ (CH_2_ scissors bending) and 1650 cm^−1^ (C=C stretching), with the ratio of these two band intensities (I1650/I1440) commonly serving as a marker for assessing the ratio of total unsaturated fatty acids to total fatty acids (Table 3). Compared with proteins, nucleic acids, and lipids, the analysis of carbohydrates using Raman spectroscopy is less common, as the spectra of saccharides, especially polysaccharides are typically complex and challenging to interpret directly. Hence, Table 4 focuses on assignments for basic monosaccharides, such as glucose, as well as the glycosidic linkage among various numbers and types of monomers in all polysaccharides.

Notably, Raman scattering of biomolecules not only reflects the identities of those molecules but also their conformational and functional properties. For instance, the secondary structures of proteins can be analyzed by examining their Amide I and III vibrations to determine the proportions of α-helix and the β-sheet structures (Table 1) [6]. Cell functions at the subcellular level and their variations with changes in temperature, pH, and ionic strength can also be investigated through Raman shifts [7]. The ability of Raman spectroscopy to probe these complex properties makes it a sensitive and selective, label-free tool in biomedicine. For further reading, readers are encouraged to consult comprehensive articles on Raman band interpretation [6–11], databases of pure biomolecules [12,13], and additional reviews on the applications of Raman spectroscopy in investigating immune cells [14–16].

### Early works in immune cell biomarkers through resonance Raman spectroscopy

The examination of immune cells through Raman spectroscopy commenced in the early 1990s, pioneered by Puppels et al. [17–19] at the University of Twente. Their work began with the first utilization of confocal Raman micro-spectroscopy to investigate a single human eosinophilic granulocyte [17]. Notably, resonance Raman spectroscopy (RRS) emerged as a cornerstone in the early investigations, extensively employed to study molecules featuring absorption bands in the visible electromagnetic spectrum. In this technique, the intrinsic low Raman scattering of molecules is significantly amplified by aligning the excitation wavelength with the absorption characteristics of the molecules of interest. Most early works on immune cells using RRS employed 532-nm green lasers to explore resonant molecules with absorption bands that overlap the excitation wavelengths, such as heme proteins (500–550 nm) or carotenoids (450–500 nm) (Table 5). Consequently, the resulting Raman resonance spectrum exhibits distinctive bands, providing heightened selectivity for the specific chromophore under investigation [20].

Neutrophils, in phagocytosis, exhibit a respiratory burst mediated by NADPH oxidase, generating superoxide within the vacuole for microbial killing [21]. RRS benefits the study of cytochrome *b*_558_ (cyt *b*_558_), the catalytic core of NADPH oxidase, due to the resonance effect of its heme group [22]. Early RRS studies in 1991 detected cyt *b*_558_ in neutrophils, revealing heme's states and redox changes [23]. Studies with PMA activation showcased cyt *b*_558_ and myeloperoxidase changes, especially in neutrophil subsets [24,25]. Raman micro-spectroscopy further mapped subcellular distributions of cyt *b*_558_ in neutrophils and eosinophil peroxidase in eosinophils [26,27].

Notably, in addition to directly identifying and mapping cytochromes, recent studies utilizing RRS have delved into examining the redox states of cytochromes and the overall metabolic condition of mitochondria. The Raman spectra of oxidized and reduced cytochromes are significantly different. RRS enables the distinction between cytochromes’ redox states, thereby aiding in the study of molecular dynamics within isolated mitochondria [28–30], apoptotic cells [31], cancer cells [32], and glutamate-stressed neuronal cells [33]. For instance, a high concentration of reduced cytochrome c has been linked to mitochondrial dysfunction and the severity of cancer. Overall, RRS provides changes in conformational states and real-time insights into mitochondrial metabolism, making it a valuable tool for evaluating mitochondrial activity and dysfunction, as well as for the continuous study of the electron transport chain.

Carotenoids, besides heme proteins, were explored in immune cells through RRS [34] (Table 5). Recognized for their role in immunoreactivity modulation and potential anti-cancer effects, carotenoids were found in serum and accumulated in human lymphocytes [19]. Studies by Puppels et al. revealed the uptake of extracellular beta-carotene by lymphocytes and its transportation to the Golgi bodies *in vitro* [19]. Recent research mapped the subcellular the distribution of carotenoids in individual lymphocytes with high sensitivity, corresponding to 415 molecules per voxel [36].

### Identifying and classifying immune cells with their Raman fingerprints

The journey into understanding immune cells using the full fingerprint using Raman micro-spectroscopy commenced with an early investigation of human neutrophilic, eosinophilic, and basophilic granulocytes [18], paving the way for subsequent advancements in chemometric analysis and machine learning classification models and their routine uses in Raman studies. J. Popp and colleagues at Jena University significantly contributed to this endeavor, first demonstrating accurate blood cell identification in cerebrospinal fluid through the synergy of fluorescence labeling and Raman spectroscopy [37]. Subsequently, they differentiated subpopulations of white blood cells entirely based on their Raman spectra, including leukocytes, leukemic cells, and solid tumor cells, demonstrating the power of classification models [38,39]. More recently, the authors utilized fluorescence, Raman, and coherent anti-Stokes Raman scattering (CARS) microscopy to characterize the inflammatory process in cells. Unstimulated eosinophils, compared with isolated eosinophils, exhibited distinctions in lipid bodies, nucleus morphology, and the absence of eosinophilic peroxidase [40]. High-throughput Raman spectroscopy, validated by flow cytometry, further identified a subpopulation of T cell receptor (TCR)αβ^+^ CD4^+^ T cells in small intestinal intra-epithelial lymphocytes from T cell-transferred mice [41]. The identification of immune cell populations is also possible through specific spectroscopic markers, for example, characteristic β-carotene bands exclusively found in T lymphocytes compared with B lymphocytes [42].

Cell activation stands as a pivotal and early phase in initiating antigen-dependent or independent stimulation of both adaptive and innate immune cells. This activation initiates signaling cascades as a defence mechanism, with the subsequent intracellular signaling events ultimately lead to morphological and molecular changes [43]. Through Raman micro-spectroscopy and machine learning, label-free characterization of immune cell activation has been investigated [25,44–46]. In a comprehensive study, Chaudhary et al. [46] studied activated and resting leukocyte subtypes, including T cells activated with phytohaemagluttinin, B cells activated with PMA or IL-4 and CD40 ligand, and monocytes activated with PMA or lipopolysaccharide. Classification models and spectral fitting analysis unveiled potential spectral biomarkers associated with the activation states of immune cells, including apotransferrin, arachidonic acid, carbonic anhydrase, DNA, histone, linolenic acid, phosphatidylcholine, among others. These studies demonstrate that the Raman fingerprint approach promises deeper insights into the intricate world of immune cell dynamics during activation.

### Decoding autoimmune diseases and disease diagnosis

Autoimmune diseases, a spectrum of conditions marked by aberrant immune responses to the body's own components, affect a diverse demographic and a higher prevalence in women [47]. Previously deemed rare, extensive epidemiological studies now reveal a substantial impact of autoimmune disorders, affecting 3–5% of the population [48]. Among these conditions, autoimmune thyroid disease and type I diabetes emerge as the most common, with others include rheumatoid arthritis (RA), multiple sclerosis (MS), Sjögren's syndrome (SS), systemic lupus erythematosus, Hashimoto's thyroiditis and psoriasis. Myalgic encephalomyelitis/chronic fatigue syndrome (ME/CFS), Fibromyalgia, and unexplained post-acute infection syndromes (PAISs), including long COVID, are characterized by systemic exertion intolerance that manifests mainly as neurological and immunological symptoms and is accompanied by chronic fatigue that is not relieved by sleep or rest. ME/CFS and PAISs, although largely unexplained and under studied, are proposed to be caused by autoimmune activation resulting either from the immune system trying to target the pathogen or from bystander autoimmune activation unrelated to pathogen structure [49]. Diagnosis of these autoimmune disorders presents a challenge, given the overlapping symptoms that frequently lead to the transformation of one disease into another. Furthermore, the underlying mechanisms contributing to the dysregulation of the immune response remain inadequately understood, and existing diagnostic approaches often lack the capability to distinguish between different autoimmune disorders.

Raman spectroscopy holds the potential to be a quick, label-free, and unbiased analytical approach in the clinical realm. Analyzing biofluids, cells, or tissues from patients through spectroscopy provides diagnostic and monitoring potential for future healthcare, allowing for swift disease diagnosis through specific spectral markers or signatures. Blood components, such as serum and plasma, as well as cells like peripheral blood mononuclear cells (PBMCs), serve as ideal diagnostic mediums due to their ease of collection, minimal invasiveness, low cost, and routine use in clinical biology. Raman spectroscopy has demonstrated potential in the early diagnosis and classification of various autoimmune disorders [50]. A study compared RA diagnostic methods, including monitoring C-reactive protein and rheumatoid factor with a newly developed Raman spectroscopic diagnostic method, revealing significantly better overall results with a specificity of 96%, sensitivity of 88%, and correct identification of 92% of RA and healthy individuals [51]. Xu et al. [52] proposed a new potential biomarker, phenylalanine, for the diagnosis of ME/CFS using PBMCs and single-cell Raman spectroscopy. More recently, the same group of authors developed a more powerful single-cell Raman platform, combined with artificial intelligence, to analyze blood cells from 98 human subjects, comprising 61 ME/CFS patients with varying disease severity and 37 healthy and MS patients as disease control [53]. The results demonstrated that Raman profiles of blood cells can accurately distinguish between healthy individuals, disease controls, and ME/CFS patients with high accuracy (91%). Furthermore, the platform could differentiate between mild, moderate, and severe ME/CFS patients with an accuracy of 84%. The study identified specific Raman peaks that correlated with ME/CFS phenotypes, offering potential insights into biological changes and supporting the development of new therapeutics. Another study of blood-diagnosis of ME/CFS achieved perfect classification by analzing a collective set of data including demographic, blood analytic, PBMC miRNAs, extracellular vesicle (EV) miRNAs, and EV feature for 15 severe ME/CFS females and 15 healthy subjects [54].

Using tissues of pathological minor salivary glands, Xue et al. [55] established a diagnostic model based on Raman spectra for primary SS patients. The analysis revealed significant differences in biochemical molecular composition, indicating increased levels of proteins, nucleic acids, and keratin, along with a decrease in lipids in primary SS samples compared with control samples. The diagnostic model, employing principal component analysis and discrimination function analysis, achieves high sensitivity (above 91%), specificity (above 92%), and an overall accuracy of 92.4%.

## Emerging Raman technologies: potential in the future of immunometabolism

**Figure 2.**
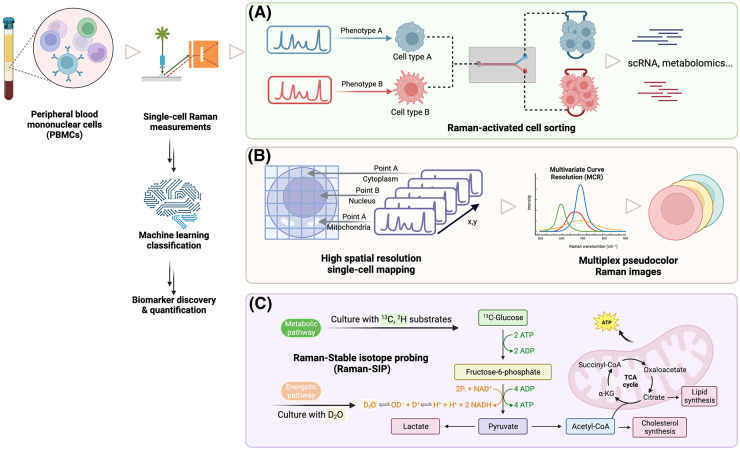
Applications of Raman technologies in immunometabolism using PBMCs as an example target. (**A**) RACS isolates and sorts individual cells guided by their Raman phenotypes; the sorted cells can be used for downstream analysis such as single-cell RNA sequencing and metabolomics. (**B**) Raman mapping allows single-cell chemical maps obtained with high spatial resolution and chemical specificity; Raman bands overlapped with each other can be resolved to individual components via multivariate curve resolution (MCR). (**C**) In Raman-SIP, cells are cultured with stable isotope-labeled substrates such as ^13^C-glucose to probe specific metabolic pathways, or heavy water (D_2_O) to probe general energetic pathways.

This section highlights three Raman technologies and their potential in unraveling the intricacies of immunometabolism at a single-cell level: Raman-activated cell sorting (RACS), Raman stable isotope probing (Raman-SIP), and Raman imaging (Figure 2). The continuous evolution of these Raman technologies holds immense promise for advancing our comprehension of immune cell dynamics, paving the way for novel applications in biomedicine. This mini-review seeks to introduce these Raman technologies to a broader audience and shares insightful perspectives on their potential for studying immunometabolism, encouraging further exploration in this rapidly evolving field.

### Raman-activated cell sorting

Fluorescence-activated cell sorting (FACS) has greatly advanced our understanding of biological systems, particularly in immunophenotyping and the detailed analysis of immune cell interactions, thanks to monoclonal antibodies [56]. While FACS bridges the gap between population, cellular, and genetic analyses, its reliance on fluorescent probes can interfere with cell metabolism and introduce immunogenicity, limiting its application in, for example, *in vivo* cell therapies using induced stem cells [57] and chimeric antigen receptor T cells [58].

RACS offers a compelling alternative, enabling label-free phenotyping by directly measuring molecular vibrations within cells. Early applications of RACS in immune cells were mostly based on optical tweezers to trap a single cell by a laser beam [37,59,60]. A more recent study by Wu et al. [61] integrated surface-enhanced Raman scattering (SERS) nanoprobes into a microfluidic system to monitor real-time communication between cancer and immune cells. Furthermore, a quantitative SERS immunoassay was conducted to assess the effectiveness of drugs in controlling the secretion patterns of cancer cells and the activity of immune cells. Recent advancements in RACS have notably broadened the versatility of RACS technologies, enhancing speed, throughput, and integrating multiple modalities [62–69]. Emerging technologies, including Raman-activated cell ejection [63,63,70] and Raman image-activated high throughput cell sorting [65] represent novel approaches yet to be employed in immune cell studies. These label-free, high-throughput single-cell sorting technologies hold great potential for revolutionizing immunometabolism by enabling precise immunophenotyping at a single-cell scale (Figure 2A).

### Raman imaging

By combining the power of optical magnification and direct visualization, Raman micro-spectroscopy can probe biological systems at a subcellular resolution. After outlining the advantages of Raman micro-spectroscopy, a significant challenge arises from the inherently weak Raman scattering, with only ∼1 in 10^7^ photons undergoing inelastic scattering. To overcome this limitation, more recent advances include SERS and coherent Raman scattering (CRS) with CARS and stimulated Raman scattering being two prominent CRS processes [71–73]. These approaches significantly enhanced the Raman signal and empower Raman technologies to high spatial resolution and multiplex Raman images (Figure 2B). Through multiple Raman modalities and digital imaging technology, Raman imaging facilitates the visualization of chemical composition and molecular structure in two or three-dimensional spaces. Each pixel in an image encapsulates chemical data specific to the molecular species present, offering a comprehensive view.

For further exploration, readers are encouraged to refer to comprehensive review articles on Raman imaging and their diverse biological and biomedical applications [74–77]. Currently, most Raman imaging employs a label-free approach, directly visualizing molecular distribution and concentrations based on intrinsic Raman bands. Another developing approach utilizes stable isotope-labeled substrates or bioorthogonal probes such as nitriles (C≡N) and carbonyls (C=O) to generate bands with high specificity in the silent region, as discussed in the next section. While the label-free approach is popular for its simplicity, it often compromises detection specificity due to the overlap of Raman bands among various biomolecular species sharing similar chemical bonds. To address this, multiple curve resolution is commonly employed to decompose the spectroscopic data (Figure 2B), from a global analytical signal, into uniquely resolved pure profiles of each contributing components [78]. Similarly, vertex component analysis handles volumetric hyperspectral datasets measured from a *z*-stack of Raman images, and can be used to reconstruct Raman images in three dimensions [79]. As summarized in a review of algorithms for image reconstruction in Raman studies [80], these computation methods enable both quantitative and qualitative analyses of individual components in complex biological samples.

### Raman stable isotope probing

The concept of introducing stable isotopes into Raman spectroscopy approaches was initially demonstrated in bacteria, eventually evolving into what is now known as Raman-SIP technology [81]. Huang et al. [82] illustrated that the introduction of ‘heavy’ ^13^C stable isotopes into cells, by culturing the cells with ^13^C-containing substrates, led to significant shifts in certain Raman bands within single-cell Raman spectra (SCRS). Subsequent research revealed that other stable isotopes, such as D (^2^H) and ^15^N, also induce Raman shifts at different positions in SCRS [83]. Raman spectroscopy combined with isotopically labeled molecules is used to study the specific metabolic features of cell constituents such as lipids, proteins, and nucleic acids. Here, we direct the readers to a comprehensive review of the applications of Raman-SIP to monitor intracellular metabolic activities [81].

Building on these findings, Raman-SIP technology has proven applicable to various biological models, including immune cells, uncovering insights into the metabolic activity of immunology (Figure 2C). Raman-SIP has been extensively used to investigate lipid uptake, distribution, and metabolism due to the strong signal of fatty acids in both the high wavenumber C–H and fingerprint regions. D-labeled free fatty acids such as d_31_-palmitic acid, d_33_-oleic acid, and d_8_-palmitic acid were used to image lipid metabolism in human monocytes and macrophages [84–86]. By using CARS imaging, Weeks et al. [87], showed that monocytes binding to vascular endothelial cells and platelets increased when activated by exposure to triglyceride-rich lipoprotein lipolysis products, two of the cardinal interactions involved in the development of atherosclerotic cardiovascular disease.

While Raman-SIP has reached maturity as a tool for studying cell ecology and biology, its application in immunology research remains at an early stage. Many applications showcase the technique's capabilities rather than providing specific answers to targeted questions. A key objective of this review is to introduce Raman–SIP to a wider audience and share insights into its potential for advancing the study of immunometabolism.

### Synergy of Raman technologies and future applications

It should be noted that many of the Raman technologies discussed earlier are frequently employed in various combinations (Figure 2). For example, Raman imaging and Raman-SIP can be used simultaneously to investigate subcellular the distribution of biomolecules and its metabolic pathways in real time. Cells with unique pathways, identified through Raman-SIP, can be isolated and sorted for downstream analysis via RACS. The synergy of Raman technologies with machine learning further enhances our understanding of complex Raman phenotypes, facilitating biomarker discovery and opening new avenues for comprehensive investigations in immunometabolism.

The potential applications are vast. Examples include:

(1) accurate and unaltered immunophenotyping of immune cell populations and functions based on their inherent characteristics;
(2) high-throughput screening of potential drugs by assessing their impact on immune cell behavior in real-time, aiding in the identification of novel therapeutic agents;
(3) cancer immunology to investigate immune responses to cancer cells and understanding the dynamics of tumor-infiltrating immune cells, which can have implications for cancer immunotherapy;
(4) infectious disease studies to investigate the behavior of immune cells in response to infectious agents, aiding in the development of treatments and vaccines;
(5) autoimmune disease research to study the behavior of immune cells in autoimmune diseases to identify potential therapeutic targets and understand disease mechanisms;
(6) cell therapy monitoring to assess the behavior and functionality of immune cells in the context of cell-based therapies, ensuring the effectiveness and safety of these treatments.

These new Raman technologies promise to reshape our approach to immunometabolism, offering insights into the dynamic interplay of immune cells and paving the way for innovative therapeutic strategies.

**Perspective**

- Raman micro-spectroscopy, a versatile tool in scientific domains, is gaining significance in biology for its label-free, multiplex, non-contact, and non-destructive analysis.
- This review simplifies Raman assignments and showcases applications in immune cell examination, identification, activation studies, and autoimmune disease diagnosis.
- We highlight three promising Raman technologies, RACS, Raman-SIP, and Raman imaging, and encourages further exploration of these evolving technologies in the rapidly advancing field of immunometabolism.

## Open Access

Open access for this article was enabled by the participation of the University of Glasgow in an all-inclusive *Read & Publish* agreement with Portland Press and the Biochemical Society under a transformative agreement with JISC.

## Abbreviations

CARS: coherent anti-Stokes Raman scattering
CFS: chronic fatigue syndrome
CRS: coherent Raman scattering
EV: extracellular vesicle
FACS: fluorescence-activated cell sorting
ME: myalgic encephalomyelitis
MS: multiple sclerosis
PAIS: post-acute infection syndrome
PBMC: peripheral blood mononuclear cell
RA: rheumatoid arthritis
RACS: Raman-activated cell sorting
RRS: resonance Raman spectroscopy
Raman-SIP: Raman stable isotope probing
SCRS: single-cell Raman spectra
SERS: surface-enhanced Raman scattering
SS: Sjögren's syndrome
